# Effect of task-oriented training assisted by force feedback hand rehabilitation robot on finger grasping function in stroke patients with hemiplegia: a randomised controlled trial

**DOI:** 10.1186/s12984-024-01372-3

**Published:** 2024-05-14

**Authors:** Yinghua Li, Yawen Lian, Xiaowei Chen, Hong Zhang, Guoxing Xu, Haoyang Duan, Xixi Xie, Zhenlan Li

**Affiliations:** Department of Rehabilitation Medicine, First Hospital of Jilin University, Changchun, China

**Keywords:** Stroke, Hand dysfunction, Force feedback hand rehabilitation robot, Task-oriented training, Neurorehabilitation

## Abstract

**Background:**

Over 80% of patients with stroke experience finger grasping dysfunction, affecting independence in activities of daily living and quality of life. In routine training, task-oriented training is usually used for functional hand training, which may improve finger grasping performance after stroke, while augmented therapy may lead to a better treatment outcome. As a new technology-supported training, the hand rehabilitation robot provides opportunities to improve the therapeutic effect by increasing the training intensity. However, most hand rehabilitation robots commonly applied in clinics are based on a passive training mode and lack the sensory feedback function of fingers, which is not conducive to patients completing more accurate grasping movements. A force feedback hand rehabilitation robot can compensate for these defects. However, its clinical efficacy in patients with stroke remains unknown. This study aimed to investigate the effectiveness and added value of a force feedback hand rehabilitation robot combined with task-oriented training in stroke patients with hemiplegia.

**Methods:**

In this single-blinded randomised controlled trial, 44 stroke patients with hemiplegia were randomly divided into experimental (*n* = 22) and control (*n* = 22) groups. Both groups received 40 min/day of conventional upper limb rehabilitation training. The experimental group received 20 min/day of task-oriented training assisted by a force feedback rehabilitation robot, and the control group received 20 min/day of task-oriented training assisted by therapists. Training was provided for 4 weeks, 5 times/week. The Fugl-Meyer motor function assessment of the hand part (FMA-Hand), Action Research Arm Test (ARAT), grip strength, Modified Ashworth scale (MAS), range of motion (ROM), Brunnstrom recovery stages of the hand (BRS-H), and Barthel index (BI) were used to evaluate the effect of two groups before and after treatment.

**Results:**

Intra-group comparison: In both groups, the FMA-Hand, ARAT, grip strength, AROM, BRS-H, and BI scores after 4 weeks of treatment were significantly higher than those before treatment (*p* < 0.05), whereas there was no significant difference in finger flexor MAS scores before and after treatment (*p* > 0.05). Inter-group comparison: After 4 weeks of treatment, the experimental group’s FMA-Hand total score, ARAT, grip strength, and AROM were significantly better than those of the control group (*p* < 0.05). However, there were no statistically significant differences in the scores of each sub-item of the FMA-Hand after Bonferroni correction (*p* > 0.007). In addition, there were no statistically significant differences in MAS, BRS-H, and BI scores (*p* > 0.05).

**Conclusion:**

Hand performance improved in patients with stroke after 4 weeks of task-oriented training. The use of a force feedback hand rehabilitation robot to support task-oriented training showed additional value over conventional task-oriented training in stroke patients with hand dysfunction.

**Clinical trial registration information:**

NCT05841108

## Background

Stroke is a leading cause of morbidity worldwide and the primary cause of motor impairment [1]. More than 80% of stroke patients with hemiplegia experience hand dysfunctions, which not only affects the use of their arms and hands in activities of daily living (ADL), but also limits their participation in social life and quality of life [2, 3].

Being the basic function of the hand, grasping plays a very important role in the activities of daily life. Simple functional activities of daily living, such as eating, dressing, grooming, and drinking, rely on the grasping function of the fingers [4]. However, grasping is a complex process that requires proper grasping force and motor control ability. When grasping, it is necessary to gradually open the fingers to form an appropriate configuration of the target object (“preshaping”). The fingers then continue to open wider than the size of the target object and stop opening at approximately 60–70% of the movement, after which they enclose the object, and finally contact its surface for grasping with appropriate force [5]. However, the grasping force and hand motor control ability are often insufficient in stroke patients, which seriously reduces the quality of movement when grasping objects in activities of daily life. It seems that finger grasping training is particularly important for improving the ability of daily living in stroke patients with hand dysfunction.

Rehabilitation therapy is considered the foundation of stroke treatment to improve the motor skills and quality of life of survivors [6]. Furthermore, repetitive training is an effective method to facilitate recovery from stroke and assist in restructuring neural networks. As a newer rehabilitation method, hand rehabilitation robots are potential tools for stroke rehabilitation treatment because they can support stable and consistent training with highly repetitive movements compared with conventional therapy [7]. However, the commonly used hand function rehabilitation robots in clinical practice are typically based on the spatiotemporal tmovement trajectory predefined by the robot computer control system, allowing patients to passively complete repeated training without requiring their active contribution, resulting in low active participation of patients [8]. A bigger problem is that most rehabilitation robots still do not apply effective input and feedback channels of sensorimotor information. In this kind of robot training, patients can only rely on visual feedback to judge the object’s size and weight to be grasped, and lack other available sensory stimuli and feedback, which affects their movement adjustment and motor control, and is not conducive to completing more accurate grasping movements [9].

Force feedback rehabilitation robots can compensate for these defects. It is a new generation of rehabilitation robots based on force feedback technology. When the wearer begins to grasp an object, information from the tactile sensors determines how much additional force the wearer needs to grasp the object, and the glove ‘strengthens’ the hand accordingly [10]. On the one hand, it can apply proportional compensation to assist the patient in completing grasping movements. On the other hand, it can provide effective force feedback information for patients, so that they can further adjust their movements according to the feedback information to achieve more accurate grasping movements. Previous studies have shown that force feedback hand rehabilitation robot training improves grip strength and hand performance in patients with spinal cord injury, articular rheumatism, and other diseases, as well as in older adults [10]. Therefore, using force feedback hand rehabilitation robots for finger grasping training in stroke patients with hemiplegia is expected to be an effective method for improving their subjective initiative and grasping function.

In addition to repetitive exercise training, another requirement for successful rehabilitation is a goal-oriented and task-specific training program to help patients use the affected side and voluntarily perform motor functions, and there are a variety of physical intervention approaches [11]. Of those, task-oriented training has been reported to be effective in improving the functional motor skills required to perform ADLs in stroke patients [12]. Task-oriented training is a therapeutic model based on the systems theory of motor control, which uses a functional approach in rehabilitating neurological patients and teaches task-specific strategies to help them adapt to changing environments [13]. This approach involves having patients practice a skill essential for achieving the goal of a task to facilitate problem-solving by enhancing their ability to adapt to various situations and developing an effective reward strategy [14–16]. In addition, for maximal learning, the approach involves behaviourally motivating patients using tasks related to their daily lives and emphasising the interaction between patients and their environment. Van Peppen et al. stated that repetitive and focused task-oriented training improved the recovery of upper limb function and enhanced motor patterns, dexterity, and agility in the upper limb [17]. The treatment effects of task-oriented training methods for stroke-related limb dysfunction have been widely recognised and supported by authoritative guidelines and systematic reviews [18, 19].

Based on the characteristics of the force feedback hand rehabilitation robot and the task-oriented training method, this study combined them to explore the effectiveness and added value of the combination of force feedback hand rehabilitation robot and task-oriented training to provide an effective rehabilitation treatment method for the recovery of hand function in stroke patients with hemiplegia and to provide a reference for the clinical application of relevant force feedback hand rehabilitation robots.

## Methods

### Study design and approval

This single-blind, parallel-group, randomised controlled trial was performed at the Department of Rehabilitation Medicine of the First Hospital of Jilin University, Jilin, China. The experimental protocol for this trial is registered at www.clinicaltrials.gov, (identifier number: NCT05841108). The local ethics committee approved the experimental protocol (22K065−001). Each participant enrolled in the trial signed a consent form.

This study used simple randomisation to create a randomisation sequence using Stata 9.0 (StataCorp, College Station, TX, USA) statistical software with a randomisation ratio of 1:1. The sample size was determined after a power calculation based on the results of a robot-based intervention that used the same training method as that used in this study [3].

### Participants

Here, we recruited patients from the Department of Rehabilitation Medicine of the first hospital of Jilin University in China. The following inclusion criteria were used to select the participants in this study: (1) first-ever stroke, (2) aged 20–80 years old, (3) post-stroke time ≤ 6 months, (4) clinically diagnosed with a central paresis of the right arm/hand (Brunnstrom stage of the affected upper limb ≥ II, Brunnstrom stage of the affected hand II-V, active flexion of the distal interphalangeal joints of the affected fingers (at least the thumb, middle and ring fingers) ≥ 10° [20], MAS of affected upper limb and finger ≤ 1+), (5) sitting balance ≥ Level 2, (6) no serious depression and no visual impairment, and (7) cognitive and speech abilities sufficient to understand instructions and to provide informed consent.

The exclusion criteria were as follows: (1) severe additional neurological, orthopedic, or rheumatoid impairments before a stroke which could interfere with task performance, (2) sensory disturbance of fingers, (3) severe joint pain caused by various factors affecting the functional activities of fingers, (4) complications with serious heart, lung, liver, kidney, or infection, and (5) attending another study or therapy to improve arm hand function.

Overall, 65 patients from the assigned hospitals underwent screening, and 44 were selected based on the selection criteria. Randomisation was implemented for participants who received either task-oriented training assisted by a force feedback hand rehabilitation robot (experimental group, *n* = 22) or a therapist (control group, *n* = 22), in addition to conventional upper limb rehabilitation training.

### Intervention

All patients received conventional upper limb rehabilitation therapy for 40 min/day, 5 days/week, for 4 weeks by experienced therapists with occupational therapist qualifications. The training contents include a range of motion training of upper limbs, motor control ability and coordination training of upper limbs and hands, ADL training, etc.

**Fig. 1:**
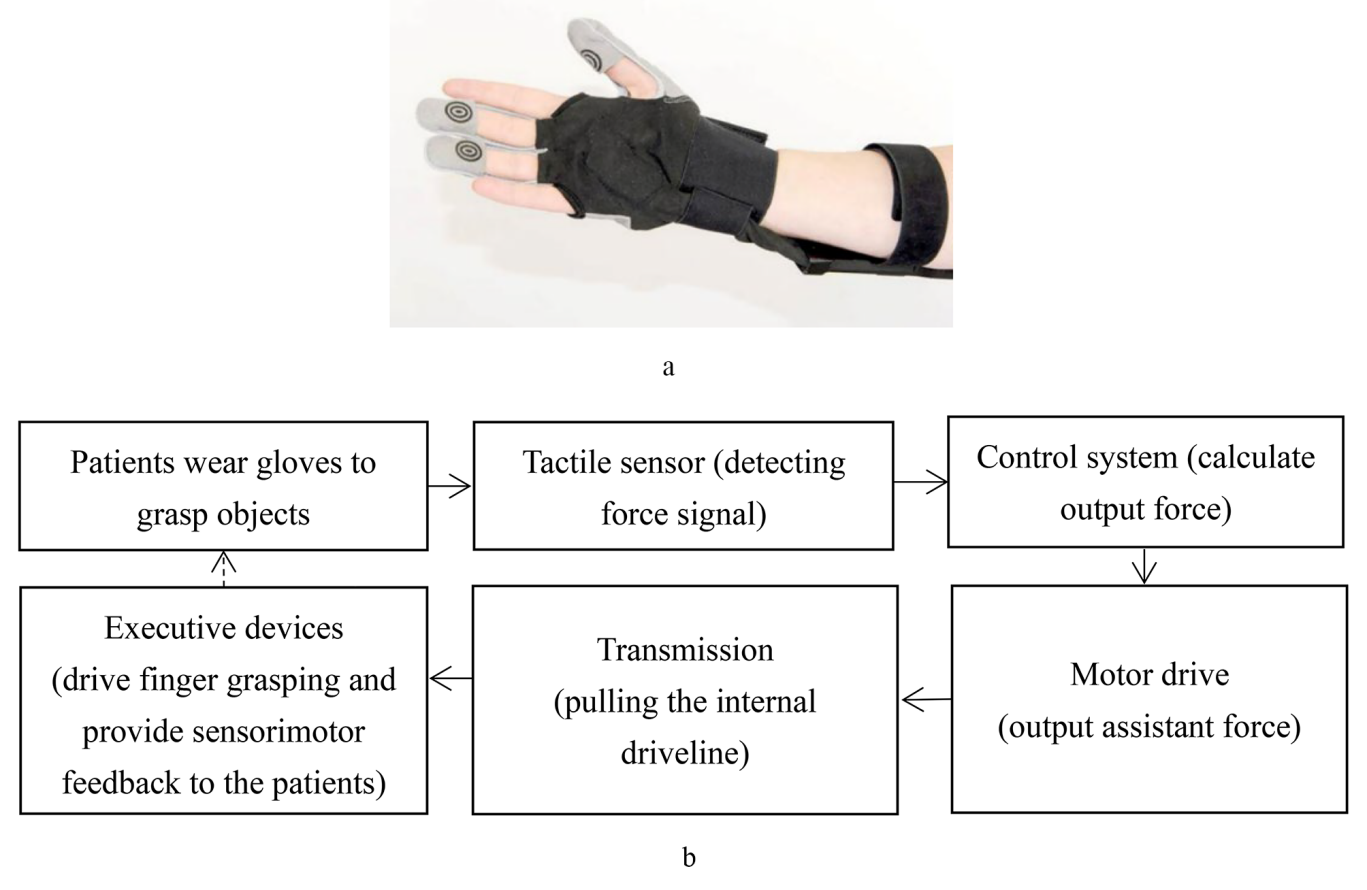
**a**. The SEM™ Glove [21]. **b**. The SEM™ Glove specific flow diagram

The experimental group received task-oriented training assisted by a force feedback hand rehabilitation robot for 20 min/day, 5 days/week, for 4 weeks. The force feedback hand rehabilitation robot used in this study is SEM™ Glove (Bioservo Technologies AB, Sweden), which can assist the patient in completing the grasping movement (Fig. 1a). It is a servo device that uses artificial tendons attached to the sides of the thumb, middle, and ring fingers. These tendons are connected to electrical motors, which actuate the thumb and finger movements by creating pulling forces. The control system used a control algorithm to calculate the ratio of finger flexion strength based on signals from tactile sensors located at the tips of the thumb, middle finger, ring finger, and palm. The device detects the intention to grip or manipulate an object via tactile sensors and applies proportional finger flexion strength to facilitate a strong grip. During this process, the patient’s finger senses the reaction force, thus providing the patient with sensorimotor feedback, based on which the patient can further adjust their movement to achieve a more accurate grasp (Fig. 1b) [20, 21].

**Fig. 2:**
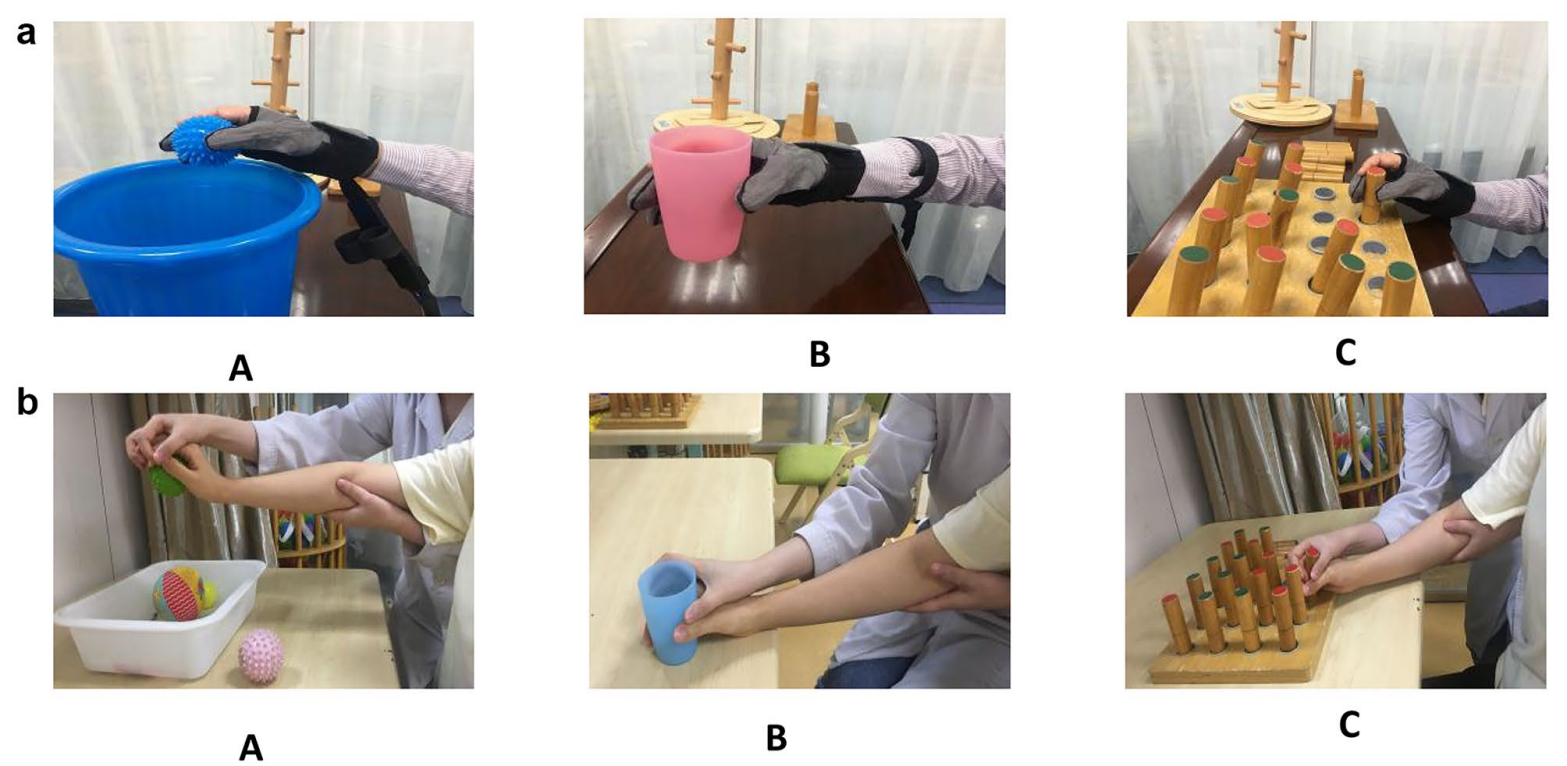
**a** Task-oriented training assisted by a force feedback rehabilitation robot (SEM™ Glove, Sweden). **A**: Grasping a ball into a barrel. **B**: Drinking water exercise. **C**: Inserting pegs. **b**. Task-oriented training assisted by a therapist. **A**: Grasping a ball into a barrel. **B**: Drinking water exercise. **C**: Inserting pegs

The therapists were asked to illustrate and demonstrate the requirements and standards for cylindrical and spherical grasp movements in the experimental group. The patients were instructed to imitate them with the non-paralytic hand and then wore SEM™ Glove to perform task-oriented training related to cylindrical and spherical grasping, such as inserting pegs, grasping a ball into a barrel, and drinking water exercises (Fig. 2a). The difficulty of task-oriented training can be adjusted according to the patient’s actual condition, such as changing the shape, weight, and size of the target, or changing the distance and duration during training. The therapist helped the patient extend their finger once they could not release the object because the glove had no extension assistance function.

The control group received task-oriented training assisted by a therapist for 20 min/day, 5 days/week, for 4 weeks to complete the same types and numbers of tasks as the experimental group. Therapists must instruct patients to try to grasp items and provide appropriate assistance to guarantee the completion of the grasping task. If finger extension is weak, the therapist assists the patient in extending of the digits before grasping the items. If the finger flexion angle does not meet the grasp function needs, the therapist should assist in flexion finger movements (Fig. 2b).

### Outcome measures

The demographical data obtained from the medical files included age, sex, date, type of stroke, hemiparetic side, and hand dominance. A blinded therapist, who was not involved in the participant selection process administered the Fugl-Meyer motor function assessment of the hand part (FMA-Hand), action research arm test (ARAT), grip strength, Modified Ashworth scale (MAS), range of motion (ROM), Brunnstrom recovery stages of the hand (BRS-H), and Barthel index (BI) before and after the 4-week intervention.

The Fugl-Meyer motor function assessment is a reliable and valid test for assessing motor function in patients with stroke [22]. This study used the Fugl-Meyer motor function assessment of the hand to evaluate the different grasping functions of the hand, with seven sub-items, scored 0–2 points, and the total score was 14 points.

The action research arm test (ARAT) has proven to be a reliable, valid, and sensitive instrument for upper limb activity measurements [23], that evaluates the ability of the hand to grasp objects of different sizes, weights, and shapes. The maximum ARAT score was 57.

The grip strength of the dominant hand was tested using an isometric hand dynamometer in the testing position recommended by the American Society of Hand Therapists (ASHT). The participants gripped the dynamometer as hard as possible without jerking. The best score from three consecutive trials was used for the analysis. Sufficient time was allowed for the participants to recover from the fatigue related to grip testing [24, 25].

The Modified Ashworth scale (MAS) was used to rate muscle tone and stiffness during passive movement of the finger flexors. The scale ranges from ‘0 = normal’, ‘1’, ‘1+’, ‘2’, ‘3’, and ‘4 = worst’ [26].

The range of motion (ROM) was measured using a goniometer. The ROM was obtained by measuring the sum of the maximum flexion of the metacarpophalangeal and interphalangeal joints of the thumb and the metacarpophalangeal, proximal, and distal interphalangeal joints of the other four fingers (summed after measuring each joint angle separately) minus the sum of the limited extension of these joints. The AROM of the affected hand was measured first, followed by the PROM. The patient was required to maintain elbow flexion, radioulnar joint pronation, a neutral wrist, and naturally extended fingers [27]. The patient was asked to flex the finger from the initial position to the maximum range to measure the flexion AROM. When measuring the extension AROM, the patient was asked to extend the finger from the naturally extended position of the hand to the maximum range. PROM was measured by the therapist when the finger was passively extended or flexed to its maximum range. Before each measurement, the therapist stretched the patient’s finger once to reduce muscle tone disturbance.

The Brunnstrom recovery stages of the hand (BRS-H) classify motor function into six levels based on recovery stages from a flaccid limb to near-normal and normal movement and coordination [28]. Higher levels indicated better motor function. In this study, levels I-VI of motor function were assigned a score from 1 to 6.

The Barthel index (BI) consists of 10 items with scores ranging from 0 to 100 and is used to assess the degree of activity and participation [29]. This study selected 5 items closely related to hand function, including feeding, grooming, toilet use, bathing, and dressing, for a total score of 40.

### Statistical analysis

Statistical analysis was performed using SPSS software (version 26.0; IBM Corp., Armonk, NY, USA). Baseline differences between the characteristics of patients in the experimental and control groups were compared using the t-test, Wilcoxon rank-sum test, or chi-square test. Changes in the clinical outcome measure scores after training were analysed using the t-test or Wilcoxon rank-sum test. Measurement data were expressed as mean ± standard deviation if normally distributed, and median (interquartile range) if not normally distributed. Count and rank data are presented as total numbers. The Bonferroni correction was used for the FMA-Hand sub-items, with *p* < 0.0071 (0.05 ÷ 7). The statistical significance of all other tests was set at *p* < 0.05. The MAS scores of 0, 1, 1+, 2, 3, and 4 were mapped as 0, 1, 1.5, 2, 3, and 4, respectively, for all statistical calculations as suggested by Rong et al. [30].

## Results

### Study participation

**Fig. 3:**
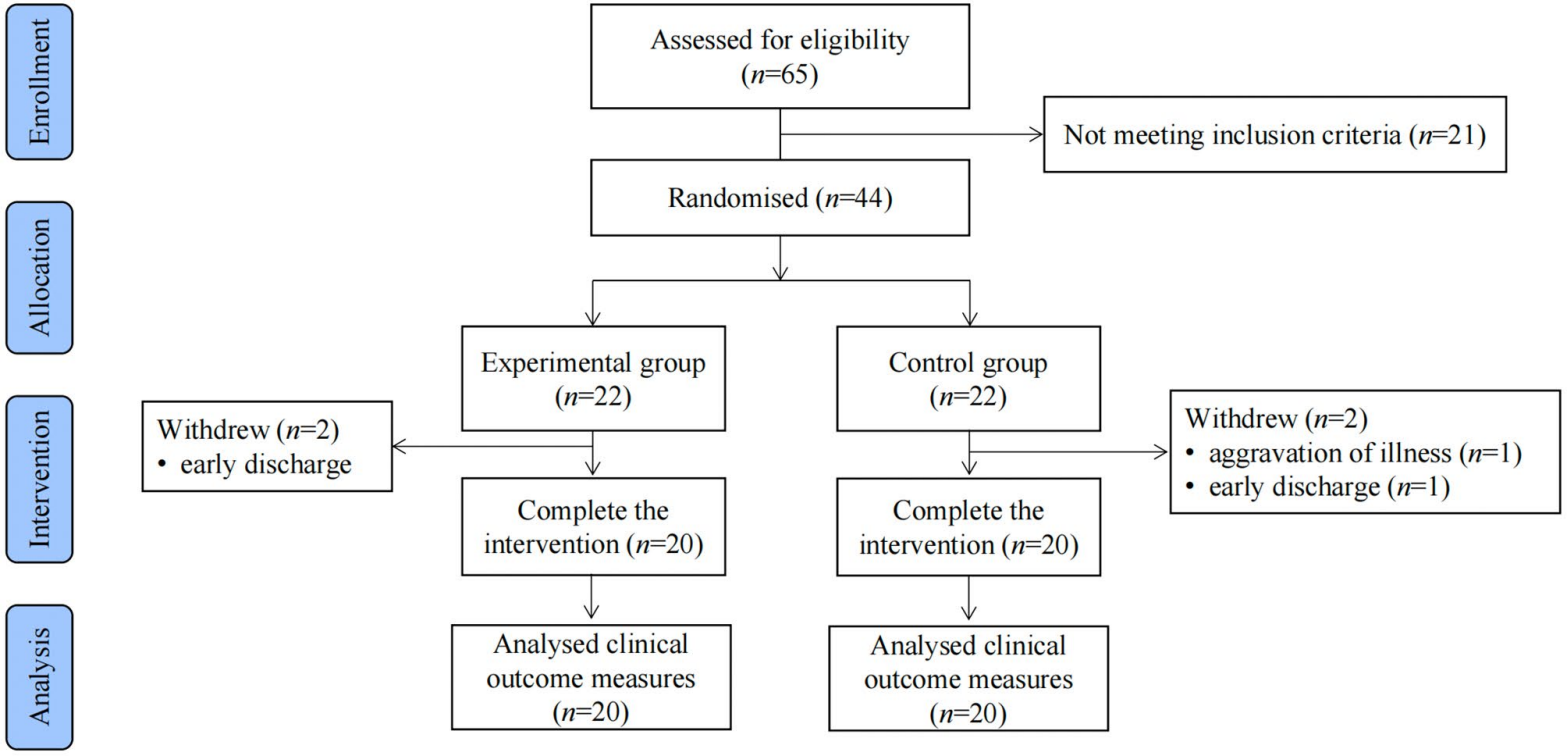
Flow diagram of the randomised controlled trial

As shown in the flow diagram in Figs. 3 and 65 patients were screened, 21 of whom did not meet the inclusion criteria and were excluded from the study. A total of 44 participants were enrolled in the study and randomised according to an allocation ratio of 1:1 to the experimental group (*n *= 22) and the control group (*n *= 22). One participant in the control group had health issues unrelated to the study and could only attend one session during the week, while one participant in the control group and two participants in the experimental group were discharged and dropped out of the study. Consequently, these data were excluded from the analysis. Therefore, data from 40 participants (20 in the control group and 20 in the experimental group) were included in this study. The study ended when all participants completed the intervention.

The final sample consisted of 33 men and 7 women, with a mean age of 63.6 ± 10.3 years and a mean time since onset of 68.95 ± 47.75 days (Table 1). 2 participants presented with hemorrhagic stroke, and 38 presented with ischemic stroke. No significant differences were found between the groups regarding demographic (sex and age) or clinical (etiology and time since injury) data at baseline. No adverse effects were detected in either the experimental or control groups.

**Table 1:** Characteristics of the participants

	Control group (*n* = 20)	Experimental group (*n* = 20)	Intergroup *P*-value
Sex (*n*, %)			NS (*p* = 0.677)
Male	17(85.0%)	16(80.0%)	
Female	3(15.0%)	4(20.0%)	
Age (years)	63.7 ± 9.4	63.6 ± 11.4	NS (*p* = 0.964)
Etiology (*n*, %)			NS (*p* = 1)
Ischemic stroke	19(95.0%)	19(95.0%)	
Hemorrhagic stroke	1(5.0%)	1(5.0%)	
Time since injury (days)	70.55 ± 49.37	67.35 ± 47.31	NS (*p* = 0.892)

Sex and etiology were analysed using the chi-square test and expressed as a percentage of the total number of participants. Age and time since injury were analysed using the t-test and expressed as mean and standard deviation. NS non-significant

### Clinical outcomes

Table 2 reports all observed changes in clinical outcome measures after treatment compared to the baseline values measured before treatment. No significant differences were detected in the clinical scales at baseline.

Both groups showed significant improvements. More in detail, all enrolled patients, regardless of treatment, showed significantly improved FMA-Hand, ARAT, grip strength, AROM, BRS-H, and BI scores (*p* *<* 0.05), whereas the MAS score did not significantly change after therapy (*p* > 0.05).

As regards the inter-group comparison, after 4 weeks of treatment, FMA-Hand total score, ARAT, grip strength, and AROM of the experimental group were better than those of the control group, with statistically significant differences (*p* < 0.05). In the FMA-Hand sub-item analysis, the scores for cylindrical and spherical grasps in the experimental group were better than those in the control group (*p* = 0.031 and *p* = 0.015, respectively). However, there were no statistically significant differences in the scores of each sub-item of the FMA-Hand after Bonferroni correction (*p* > 0.007). In addition, there were no statistically significant differences in MAS, BRS-H, and BI scores (*p* > 0.05).

**Table 2:** Changes in clinical outcome measures

Groups Outcomes		Experimental group (*n *= 20)					Control group (*n *= 20)				Intergroup *P*-value(baseline)	Intergroup *P*-value(Post-therapy)
		Pre-therapy	Post-therapy	Difference value	Intragroup *P*-value		Pre-therapy	Post-therapy	Difference value	Intragroup *P*-value		
FMA-Hand Total score		6.00(3.50,8.00)	9.00(8.00,12.75)	4.00(3.00,5.00)	^*^<0.001		6.00(2.00,7.75)	9.00(6.00,10.00)	3.00(2.25,4.00)	^*^<0.001	0.543	^*^0.015
Sub-items of the FMA-Hand												
	Finger mass flexion(0/1/2)	0/17/3	0/6/14		^#^0.001		0/15/5	0/8/12		^#^0.005	0.681	0.513
	Finger mass extension(0/1/2)	11/7/2	7/6/7		^#^0.007		11/7/2	8/5/7		^#^0.005	1.000	0.852
	Hook grasp(0/1/2)	5/11/4	2/5/13		^#^0.001		5/14/1	2/12/6		^#^0.005	0.479	0.059
	Lateral pinch(0/1/2)	6/11/3	1/11/8		^#^0.002		7/11/2	1/14/5		^#^0.007	0.639	0.364
	Interdigital pinch(0/1/2)	11/8/1	5/7/8		^#^0.006		11/7/2	5/9/6		^#^0.004	0.890	0.665
	Cylindrical grasp(0/1/2)	6/10/4	2/5/13		^#^0.001		8/10/2	2/13/5		^#^0.007	0.373	0.031
	Spherical grasp(0/1/2)	8/8/4	2/5/13		^#^0.001		9/10/1	2/14/4		^#^0.002	0.440	0.015
ARAT		29.90 ± 16.52	39.60 ± 13.99	9.70 ± 4.47	^*^<0.001		28.75 ± 16.71	35.90 ± 14.34	7.15 ± 3.23	^*^<0.001	0.828	^*^0.046
Grip strength (N)		3.42 ± 2.19	5.60 ± 2.42	2.18 ± 0.71	^*^<0.001		3.09 ± 2.13	4.05 ± 2.36	0.96 ± 0.51	^*^<0.001	0.632	^*^<0.001
MAS(0/1/1+/2/3/4)		10/6/4/0/0/0	11/7/2/0/0/0		0.083		8/10/2/0/0/0	10/8/2/0/0/0		0.157	0.860	0.786
AROM (°)		571.00(299.50,745.75)	673.00(533.00,772.25)	135.50(79.25,225.00)	^*^<0.001		516.15 ± 231.44	599.35 ± 161.53	61.50(37.50,118.00)	^*^0.001	0.976	^*^0.027
BRS-H(Stage I/II/III/IV/V/VI)		0/5/6/5/4/0	0/2/5/4/6/3		^*^<0.001		0/4/7/4/5/0	0/2/6/4/4/4		^*^0.001	0.779	0.890
BI		20.00(15.00,30.00)	25.00(21.25,33.75)	5.00(0.00,5.00)	^*^0.001		22.50(15.00,28.75)	27.50(20.00,30.00)	5.00(0.00,5.00)	^*^0.001	0.989	0.564

Abbreviations: FMA-Hand, Fugl-Meyer motor function assessment of the hand part; ARAT, Action Research Arm Test; MAS, Modified Ashworth Scale; AROM, active range of motion; BRS-H, Brunnstrom recovery stages of the hand; BI, Barthel Index. The FMA-Hand sub-items, MAS, and BRS-H were used in the Wilcoxon rank-sum test and expressed as the total number of participants. The FMA-Hand total score, ARAT, Grip strength, AROM, and BI Data were analysed using the t-test or Wilcoxon rank sum test and presented as mean ± standard deviation, or median (interquartile range).*p < 0.05 (Except for the Sub-item of the FMA-Hand, which used Bonferroni correction with ^#^*p* < 0.0071)

## Discussion

This study investigated the effectiveness of task-oriented training assisted by a force feedback hand rehabilitation robot on finger grasping function in stroke patients with hemiplegia compared to conventional task-oriented training. Our results prove that task-oriented training assisted by a force feedback hand rehabilitation robot can provide clinically meaningful improvements in the grasping function compared to conventional task-oriented training by a therapist.

From planning to execution of the movement, the nervous system needs to accurately control the hand muscle group at an appropriate time (the start and offset of the activity) and space (the offset caused by bone attachment) to produce meaningful actions [31]. However, after stroke, because of neurological function defects in the brain, abnormal structural and functional connections in brain areas related to hand function, and damaged information transmission pathways between the hand and brain, patients are often unable to complete finger movements correctly [32]. Min et al. [33] showed that in the rehabilitation training process, a combination of sensory stimulation, including visual and tactile stimulation, can provide timely and correct behavioural guidance and feedback for patients, which is conducive to reshaping the motor perception loop. The force-feedback hand rehabilitation robot used in this study provides force tactile stimulation and timely and effective sensorimotor information feedback to help patients better adjust their finger movements and complete grasping [34, 35].

After 4 weeks of treatment, compared with the control group, the experimental group’s FMA-Hand total score and ARAT score were significantly improved (*p* < 0.05), indicating that task-oriented training assisted by the force feedback hand rehabilitation robot was better than conventional task-oriented training. Studies [36] have shown that synchronizing sensory and motor information contributes to forming a correct sensorimotor loop and promotes functional remodeling of the nervous system. When performing the motor task, the force feedback hand rehabilitation robot immediately outputs the corresponding additional force according to the actual force of the affected hand during the execution of the motor task and feedbacks the sensorimotor information to the finger of the patient to promote the integration of the patient’s subjective motor awareness and objective sensory information and build a complete sensory-motor conduction pathway to ensure the smooth completion of the task. However, conventional rehabilitation training assisted by a therapist often relies on the therapist’s verbal feedback and the assisting force provided by the therapist’s subjective judgment, resulting in patients being unable to obtain complete feelings and immediate and effective feedback, which may be one of the reasons for the additional effects of task-oriented training assisted by the force feedback hand rehabilitation robot. In this study, we compared the scores of the seven sub-items of the FMA-Hand scale. The results showed that the cylindrical and spherical grasp scores in the experimental group were slightly better than those in the control group (*p* = 0.031 and *p* = 0.015, respectively). However, these differences did not reach statistical significance after the Bonferroni correction (*p* > 0.007). It is plausible that the 4-week rehabilitation training program might be insufficient to capture such interventions’ intensity and temporal effects adequately. Surprisingly, fine motor function of the fingers, such as lateral and interdigital pinching, also improved in both groups in this study, even though only gross grasp function was practiced. Improving gross grasp will also likely benefit more intricate grip types. In essence, hand function is mediated by synergistic sets of muscles, where improvement in one synergy is likely to benefit another because muscles are shared between synergies. Thus, gross grasp training of the fingers may help improve the fine motor function of patients’ fingers [37].

Grip strength is closely related to complex tasks of the upper limb and is key to rehabilitating hand function after stroke. Even if patients have finger grasping and extension movements, insufficient grip strength can interfere with holding objects or performing daily activities. The results of this study showed that the grip strength of both groups was significantly improved after 4 weeks of treatment, and the improvement degree of the grip strength of the experimental group was better than that of the control group (△post-pre: 2.18 ± 0.71 and 0.96 ± 0.51, respectively; *p* < 0.05), indicating that although the two training methods were effective in improving the finger grip strength, task-oriented training assisted by force feedback hand rehabilitation robot was more effective. Moreover, According to the scoring criteria of the FMA-Hand and ARAT scales, finger strength had a greater impact on the scores, which also explains why the FMA-Hand total and ARAT scores of the experimental group were significantly better than those of the control group. Radder et al. [20] found that the grip strength of older people significantly improved after repeated grasping training with the assistance of a force feedback hand rehabilitation robot, which is consistent with the results of this study. Seo et al. [38] indicated that sensory stimulation during finger grasping can activate the brain’s sensorimotor cortex and promote grip strength recovery. We believe that intensive grasping training with the assistance of force feedback hand rehabilitation robots can increase the effective input of sensorimotor information and promote the recruitment of motor units and synchronisation of the activities of hand muscle groups, thereby improving the function of nerve-innervating muscles and enhancing grip strength [39]. A previous study [40] showed that active conscious rehabilitation training promotes cortical reorganisation associated with motor recovery. The force feedback hand rehabilitation robot was designed to encourage active engagement in a motor task. It can provide a corresponding proportion of additional force according to the patient’s strength, improving their active participation and strength training. This feature of the robot may be one of the important reasons why task-oriented training assisted by the force feedback hand rehabilitation robot in this study was superior to conventional task-oriented training in improving grip strength.

In patients with stroke, hand grip strength is often accompanied by increased muscle tone in the flexor muscle groups of the fingers. In this study, the grip strength and MAS results showed that the finger grip strength of the experimental group significantly improved. However, the muscle tone did not significantly change, suggesting that force feedback hand rehabilitation robot training did not adversely affect muscle tone. We believe that the input and feedback of various types of sensorimotor information during patient training can help generate correct proprioceptive signals, reduce compensatory behaviour, improve coordination between muscle groups, and inhibit abnormal increases in muscle tone [41]. The results of this study were consistent with those of a previous report by Osuagwu et al. [42], who found that patients with cervical SCI wore force feedback gloves to complete daily activities, and the muscle tone of the patient’s upper limbs did not change significantly.

An adequate ROM of the finger joints is a key factor affecting whether the hand can grasp objects smoothly. Some studies [43] found that centralised exercise and skill training can help improve the AROM of patients with stroke and promote an increase in gray matter volume in multiple brain regions, indicating that functional activities mainly focusing on grasping training can help promote recovery of hand function and further promote the activation and reorganisation of the motor cortex. This study’s result showed that after 4 weeks of treatment, finger AROM in both groups significantly improved compared with that before treatment, suggesting that both treatments were effective. The AROM results of the experimental group were better than those of the control group, indicating that task-oriented training assisted by force feedback hand rehabilitation robots can effectively improve the AROM of the affected hand, supporting the results of the FMA-Hand and ARAT scores in this study.

This study’s result showed that the BRS-H of the two groups of patients significantly improved after treatment, indicating that the two training methods effectively promoted the separation of the affected hand, improved the movement mode, and restored the finger function. Studies [44] have shown that task-oriented training such as repeated reaching, grasping, and releasing a ball can help improve the coordination of upper limb and hand movements and promote separation movements. This improvement in coordination and promotion of separation movements through task-oriented training may also be one of the reasons why the patient’s finger mass extension score on the FMA-Hand subscale improved in both groups after 4 weeks of treatment (*p* = 0.007 and *p* = 0.005, respectively). However, the results of this study showed no significant difference in BRS-H between the two groups, which can be explained by two reasons: (1) the training time was relatively short, which may lead to no significant difference in the ratings between the two groups, and (2) the sensitivity of the scale is low, which makes it impossible to detect the difference between the two groups.

The ADL ability of patients with stroke decreases to different degrees, seriously affecting their quality of life and causing serious burdens to their families. Therefore, an improvement in ADL ability is an important indicator of functional recovery in patients with stroke. A previous study [45] showed that task-oriented training based on virtual reality using the Gloreha2 rehabilitation robot could improve the upper limb function and ADL ability of patients with stroke, which may be related to the real-time visual feedback and rich sensory input provided by the rehabilitation robot to stimulate sensorimotor neural networks and effectively promote neural remodeling. The results of this study showed that after 4 weeks of treatment, the BI scores of patients in both groups were significantly improved compared with those before treatment; however, there was no significant difference between the two groups, which may be related to many factors, such as the selection of study objects, sample size, treatment intensity, and time. However, some BI scale defects cannot be ruled out. For example, the BI scale tends to determine whether patients can complete activities of daily living but does not provide detailed requirements on the degree of participation of the affected hand. The score was not sufficiently refined to fully reflect the differences in ADL between the two groups.

In summary, task-oriented training assisted by force feedback hand rehabilitation robots combined with conventional therapy can effectively improve hand function in patients with stroke. The force feedback hand rehabilitation robot can provide repetitive and stable rehabilitation training for the affected hand and sensorimotor information feedback to promote the reorganisation of brain function. Additionally, the device is small, convenient to carry, simple to operate, and suitable for patients to wear daily. These qualities can reduce the financial burden on patients and save social and medical resources, which is worthy of wide clinical application and promotion.

This study has several limitations. It is impossible to understand the long-term impact of robot-assisted training on hand function recovery in patients with stroke because of the short study period and lack of follow-up studies. Moreover, because of the small sample size and insufficient stratification, we did not observe the potential impact of different ages, sexes, injury types, disease courses, and other factors on the treatment effect. Therefore, a randomised controlled study with a larger sample size and a follow-up study are needed to investigate how the level and methodology of force feedback rehabilitation robot training could impact hand function improvement. Furthermore, we need to combine EMG, fMRI, fNIRS, and other quantitative indicators to evaluate the improvement in brain and hand function in a real-time, objective, and quantitative manner to explore the rehabilitation effect and possible mechanism of force feedback rehabilitation robots on the hand function of patients with stroke. In addition, the rehabilitation robot used in this study mainly focused on finger grasping and lacked finger extension functions. Based on the experience of conducting this study, we believe that the gloves under study and similar devices will likely appeal to more people with impaired hand function if they support both hand extension and flexion.

## Conclusions

Hand performance improved in stroke patients with hemiplegia after 4 weeks of task-oriented training in both robot- and non-technology-supported interventions. However, using a force feedback hand rehabilitation robot to support task-oriented training showed additional value over conventional task-oriented training, mainly in improving finger AROM, grip strength, and hand motor function. Therefore, task-oriented training assisted by a force feedback robot may have future implications in facilitating stroke recovery. Further research should be conducted to determine the possible mechanism by which force feedback robots affect the hand movement function in patients with stroke from a deeper level.

## Data Availability

The datasets for this study are available from the corresponding author on reasonable request.

## Abbreviations

ADL: Activities of daily living
MAS: Modified Ashworth scale
ROM: Range of motion
AROM: Active range of motion
PROM: Passive range of motion
FMA-Hand: Fugl-Meyer motor function assessment of the hand part
ARAT: Action research arm test
BRS-H: Brunnstrom recovery stages of the hand
BI: Barthel index
ASHT: American Society of Hand Therapists
